# (5-Ethenyl-1-aza­bicyclo­[2.2.2]octan-2-yl)(6-meth­oxy-3-quinol­yl)methanol methanol solvate

**DOI:** 10.1107/S1600536809045073

**Published:** 2009-10-31

**Authors:** Savitha Muramulla, Hadi D. Arman, Cong-Gui Zhao, Edward R. T. Tiekink

**Affiliations:** a Department of Chemistry, The University of Texas at San Antonio, One UTSA Circle, San Antonio, Texas 78249-0698, USA; b Department of Chemistry, University of Malaya, 50603 Kuala Lumpur, Malaysia

## Abstract

In the title methanol solvate, C_20_H_24_N_2_O_2_·CH_4_O, an L-shaped conformation is found as the two substituents at the central hydr­oxy group are almost orthogonal to each other [the C—C—C angle at the central *sp* ^3^-C atom is 110.12 (13)°]. The most notable feature of the crystal packing is the formation of supra­molecular chains along the *b* direction mediated by O—H⋯N hydrogen bonds occurring between the hydr­oxy and quinoline N atoms; the methanol mol­ecules are linked to these chains *via* O—H⋯N_amine_ hydrogen bonds. C—H⋯O inter­actions also occur.

## Related literature

For background to pre-catalyst mol­ecules for the Michael addition of acetone to *trans*-β-nitro­styrene, see: Mandal & Zhao (2008 ▶).
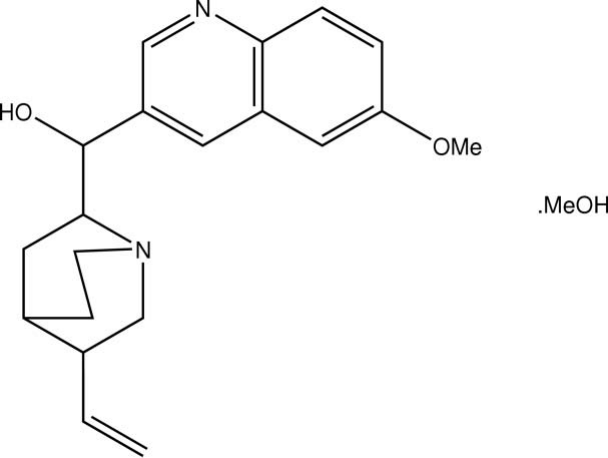


## Experimental

### 

#### Crystal data

C_20_H_24_N_2_O_2_·CH_4_O
*M* *_r_* = 356.45
Orthorhombic, 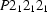

*a* = 9.5374 (13) Å
*b* = 12.9842 (17) Å
*c* = 15.871 (2) Å
*V* = 1965.4 (4) Å^3^
*Z* = 4
Mo *K*α radiation
μ = 0.08 mm^−1^
*T* = 98 K
0.12 × 0.10 × 0.04 mm

#### Data collection

Rigaku AFC12K/SATURN724 diffractometer
Absorption correction: multi-scan (*ABSCOR*; Higashi, 1995 ▶) *T*_min_ = 0.788, *T*_max_ = 1.000
14410 measured reflections
2561 independent reflections
2501 reflections with *I* > 2σ(*I*)
*R*_int_ = 0.043

#### Refinement

*R*[*F* ^2^ > 2σ(*F* ^2^)] = 0.039
*wR*(*F* ^2^) = 0.108
*S* = 1.08
2561 reflections
243 parameters
2 restraints
H-atom parameters constrained
Δρ_max_ = 0.35 e Å^−3^
Δρ_min_ = −0.27 e Å^−3^

### 

Data collection: *CrystalClear* (Rigaku/MSC, 2005 ▶); cell refinement: *CrystalClear*; data reduction: *CrystalClear*; program(s) used to solve structure: *SHELXS97* (Sheldrick, 2008 ▶); program(s) used to refine structure: *SHELXL97* (Sheldrick, 2008 ▶); molecular graphics: *DIAMOND* (Brandenburg, 2006 ▶); software used to prepare material for publication: *SHELXL97*.

## Supplementary Material

Crystal structure: contains datablocks global, I. DOI: 10.1107/S1600536809045073/hb5191sup1.cif

Structure factors: contains datablocks I. DOI: 10.1107/S1600536809045073/hb5191Isup2.hkl

Additional supplementary materials:  crystallographic information; 3D view; checkCIF report

## Figures and Tables

**Table 1:** Hydrogen-bond geometry (Å, °)

*D*—H⋯*A*	*D*—H	H⋯*A*	*D*⋯*A*	*D*—H⋯*A*
O3—H3o⋯N1	0.84	1.95	2.783 (2)	171
O1—H1o⋯N2^i^	0.84	1.92	2.751 (2)	173
C20—H20b⋯O1^ii^	0.98	2.33	3.298 (2)	171
C18—H18⋯O3^iii^	0.95	2.58	3.471 (2)	155

Symmetry codes: (i) 
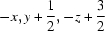
; (ii) 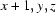; (iii) 
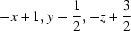
.

**Fig. 1.**
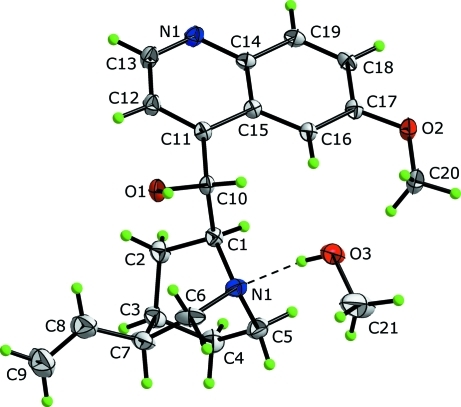
Molecular structure of the asymmetric unit in (I) showing atom-labelling scheme and displacement ellipsoids at the 50% probability level. The O—H···N hydrogen bond is shown as a dashed line.

**Fig. 2.**
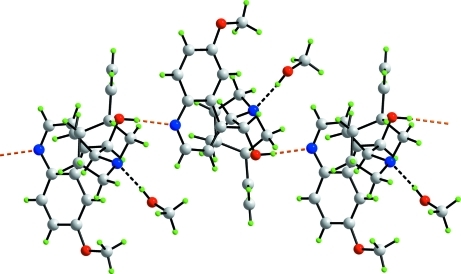
Supramolecular chain in (I) mediated by O–H···N hydrogen bonds (orange dashed lines). Methanol molecules are associated with this chain via O–H···N hydrogen bonds (black dashed lines).

## supplementary crystallographic information

### Comment

Molecules related to and including the title compound, (I), have been evaluated as pre-catalysts for the Michael addition of acetone to *trans*-β-nitrostyrene, see: Mandal & Zhao (2008).

The molecule of (I), Fig. 1, adopts an `L'-shaped conformation whereby the substituted quinoline and dabco residues are linked at the central *sp*^3^-C10 atom carrying the hydroxy group, the C1–C10–C11 angle is 110.12 (13)°. Viewed down the N1···C3 axis, the dabco molecule adopts an essentially eclipsed conformation. Both the hydroxy and vinyl groups are orientated towards the same side of the molecule.

In the crystal structure, molecules are connected into a supramolecular chain along the *b* axis *via* O—H···N2 hydrogen bonds formed between the O1-hydroxy group and the N2 atom of the quinoline residue, Table 1 and Fig. 2. The lattice methanol molecules associate with this chain *via* O—H···N1 hydrogen bonds. Chains are consolidated in the crystal packing by C–H···O interactions, Table 1.

### Experimental

Quinidine (TCI America Chemicals) and `L'-proline (Sigma Aldrich) were obtained commercially and used as received. A 1:1 molar ratio of quinidine (100 mg) and `L'-proline (35 mg) were taken in methanol (8 ml) and upon upon vapour diffusion of hexane, colourless crystals formed within 7 days.

### Refinement

The H atoms were geometrically placed (O—H = 0.84 Å and C—H = 0.95–1.00 Å) and refined as riding with *U*_iso_(H) = 1.2*U*_eq_(C) or 1.5*U*_eq_(O, methyl-C). In the absence of significant anomalous scattering effects, 1951 Friedel pairs were averaged in the final refinement.

### Crystal data

**Table:**

C_20_H_24_N_2_O_2_·CH_4_O	*F*(000) = 768
*M**_r_* = 356.45	*D*_x_ = 1.205 Mg m^−^^3^
Orthorhombic, *P*2_1_2_1_2_1_	Mo *K*α radiation, λ = 0.71073 Å
Hall symbol: P 2ac 2ab	Cell parameters from 7299 reflections
*a* = 9.5374 (13) Å	θ = 2.0–40.2°
*b* = 12.9842 (17) Å	µ = 0.08 mm^−^^1^
*c* = 15.871 (2) Å	*T* = 98 K
*V* = 1965.4 (4) Å^3^	Platelet, colourless
*Z* = 4	0.12 × 0.10 × 0.04 mm

### Data collection

**Table:**

Rigaku AFC12K/SATURN724 diffractometer	2561 independent reflections
Radiation source: fine-focus sealed tube	2501 reflections with *I* > 2σ(*I*)
graphite	*R*_int_ = 0.043
ω scans	θ_max_ = 27.5°, θ_min_ = 2.0°
Absorption correction: multi-scan (*ABSCOR*; Higashi, 1995)	*h* = −12→12
*T*_min_ = 0.788, *T*_max_ = 1.000	*k* = −16→16
14410 measured reflections	*l* = −18→20

### Refinement

**Table:**

Refinement on *F*^2^	Secondary atom site location: difference Fourier map
Least-squares matrix: full	Hydrogen site location: inferred from neighbouring sites
*R*[*F*^2^ > 2σ(*F*^2^)] = 0.039	H-atom parameters constrained
*wR*(*F*^2^) = 0.108	*w* = 1/[σ^2^(*F*_o_^2^) + (0.067*P*)^2^ + 0.3164*P*] where *P* = (*F*_o_^2^ + 2*F*_c_^2^)/3
*S* = 1.08	(Δ/σ)_max_ = 0.001
2561 reflections	Δρ_max_ = 0.35 e Å^−^^3^
243 parameters	Δρ_min_ = −0.27 e Å^−^^3^
2 restraints	Absolute structure: nd
Primary atom site location: structure-invariant direct methods	

### Special details

**Table:**

Geometry. All s.u.'s (except the s.u. in the dihedral angle between two l.s. planes) are estimated using the full covariance matrix. The cell s.u.'s are taken into account individually in the estimation of s.u.'s in distances, angles and torsion angles; correlations between s.u.'s in cell parameters are only used when they are defined by crystal symmetry. An approximate (isotropic) treatment of cell s.u.'s is used for estimating s.u.'s involving l.s. planes.
Refinement. Refinement of *F*^2^ against ALL reflections. The weighted *R*-factor *wR* and goodness of fit *S* are based on *F*^2^, conventional *R*-factors *R* are based on *F*, with *F* set to zero for negative *F*^2^. The threshold expression of *F*^2^ > 2σ(*F*^2^) is used only for calculating *R*-factors(gt) *etc*. and is not relevant to the choice of reflections for refinement. *R*-factors based on *F*^2^ are statistically about twice as large as those based on *F*, and *R*- factors based on ALL data will be even larger.

### Fractional atomic coordinates and isotropic or equivalent isotropic displacement parameters (Å^2^)

**Table:**

	*x*	*y*	*z*	*U*_iso_*/*U*_eq_	
O1	−0.09211 (13)	0.48893 (9)	0.86000 (8)	0.0208 (3)	
H1O	−0.0859	0.5528	0.8526	0.031*	
O2	0.56486 (13)	0.36533 (10)	0.77309 (8)	0.0235 (3)	
O3	0.32503 (17)	0.60866 (12)	0.92437 (9)	0.0349 (4)	
H3O	0.2607	0.5712	0.9435	0.052*	
N1	0.12984 (17)	0.48168 (12)	1.00313 (9)	0.0245 (3)	
N2	0.05966 (17)	0.19494 (12)	0.67553 (10)	0.0228 (3)	
C1	0.08927 (18)	0.39987 (13)	0.94193 (10)	0.0191 (3)	
H1	0.1754	0.3580	0.9312	0.023*	
C2	−0.0200 (2)	0.32607 (14)	0.98034 (12)	0.0250 (4)	
H2A	−0.1135	0.3393	0.9556	0.030*	
H2B	0.0061	0.2537	0.9684	0.030*	
C3	−0.0236 (2)	0.34482 (17)	1.07595 (13)	0.0299 (4)	
H3	−0.0827	0.2913	1.1041	0.036*	
C4	0.1270 (2)	0.33999 (17)	1.10976 (13)	0.0324 (5)	
H4A	0.1711	0.2738	1.0936	0.039*	
H4B	0.1266	0.3449	1.1720	0.039*	
C5	0.2101 (2)	0.43074 (17)	1.07174 (13)	0.0304 (4)	
H5A	0.2310	0.4816	1.1165	0.036*	
H5B	0.3004	0.4051	1.0491	0.036*	
C6	0.0053 (2)	0.53057 (16)	1.04185 (12)	0.0327 (5)	
H6A	−0.0539	0.5607	0.9970	0.039*	
H6B	0.0362	0.5872	1.0792	0.039*	
C7	−0.0833 (2)	0.45269 (18)	1.09371 (12)	0.0329 (5)	
H7	−0.0694	0.4681	1.1549	0.039*	
C8	−0.2379 (3)	0.4605 (2)	1.07452 (16)	0.0498 (7)	
H8	−0.2650	0.4626	1.0170	0.060*	
C9	−0.3375 (3)	0.4646 (2)	1.13121 (18)	0.0534 (7)	
H9A	−0.3145	0.4626	1.1894	0.064*	
H9B	−0.4327	0.4695	1.1141	0.064*	
C10	0.04468 (16)	0.44563 (13)	0.85620 (11)	0.0175 (3)	
H10	0.1126	0.5010	0.8402	0.021*	
C11	0.04985 (18)	0.36143 (13)	0.78993 (10)	0.0182 (3)	
C12	−0.07008 (19)	0.31535 (14)	0.76062 (12)	0.0227 (4)	
H12	−0.1593	0.3392	0.7787	0.027*	
C13	−0.06039 (19)	0.23226 (15)	0.70350 (12)	0.0249 (4)	
H13	−0.1449	0.2015	0.6842	0.030*	
C14	0.18062 (18)	0.24173 (13)	0.70160 (10)	0.0196 (3)	
C15	0.18147 (17)	0.32619 (13)	0.75884 (10)	0.0172 (3)	
C16	0.31191 (18)	0.37064 (13)	0.78240 (10)	0.0180 (3)	
H16	0.3137	0.4283	0.8193	0.022*	
C17	0.43522 (18)	0.33065 (13)	0.75211 (11)	0.0191 (3)	
C18	0.43402 (19)	0.24588 (14)	0.69502 (11)	0.0213 (3)	
H18	0.5199	0.2187	0.6743	0.026*	
C19	0.31040 (19)	0.20397 (14)	0.67016 (11)	0.0216 (3)	
H19	0.3106	0.1485	0.6311	0.026*	
C20	0.57014 (19)	0.44156 (15)	0.83814 (14)	0.0288 (4)	
H20A	0.5219	0.5040	0.8191	0.043*	
H20B	0.6682	0.4579	0.8510	0.043*	
H20C	0.5240	0.4150	0.8888	0.043*	
C21	0.3789 (4)	0.6699 (3)	0.98995 (18)	0.0793 (13)	
H21A	0.3187	0.7303	0.9979	0.119*	
H21B	0.4740	0.6925	0.9755	0.119*	
H21C	0.3816	0.6296	1.0421	0.119*	

### Atomic displacement parameters (Å^2^)

**Table:**

	*U*^11^	*U*^22^	*U*^33^	*U*^12^	*U*^13^	*U*^23^
O1	0.0171 (6)	0.0161 (5)	0.0292 (6)	0.0017 (5)	0.0012 (5)	−0.0008 (5)
O2	0.0157 (5)	0.0233 (6)	0.0314 (6)	−0.0006 (5)	0.0013 (5)	−0.0028 (5)
O3	0.0421 (9)	0.0361 (8)	0.0265 (6)	−0.0121 (7)	0.0075 (6)	−0.0059 (6)
N1	0.0303 (8)	0.0229 (7)	0.0203 (6)	−0.0048 (6)	−0.0025 (6)	−0.0006 (6)
N2	0.0230 (7)	0.0193 (7)	0.0262 (7)	−0.0012 (6)	−0.0012 (6)	−0.0032 (6)
C1	0.0206 (8)	0.0166 (7)	0.0202 (7)	−0.0011 (6)	0.0009 (6)	0.0007 (6)
C2	0.0281 (9)	0.0209 (8)	0.0259 (8)	−0.0048 (7)	0.0028 (7)	0.0031 (7)
C3	0.0292 (9)	0.0362 (11)	0.0242 (8)	−0.0031 (8)	0.0029 (8)	0.0098 (8)
C4	0.0322 (10)	0.0360 (11)	0.0291 (9)	0.0001 (9)	−0.0013 (8)	0.0110 (8)
C5	0.0320 (10)	0.0346 (10)	0.0244 (8)	−0.0049 (8)	−0.0060 (8)	0.0027 (8)
C6	0.0487 (12)	0.0262 (10)	0.0234 (8)	0.0091 (9)	−0.0014 (9)	−0.0047 (8)
C7	0.0318 (10)	0.0468 (12)	0.0201 (8)	0.0083 (10)	0.0039 (8)	−0.0020 (8)
C8	0.0360 (12)	0.080 (2)	0.0335 (11)	0.0157 (13)	−0.0006 (10)	−0.0086 (13)
C9	0.0388 (12)	0.0715 (19)	0.0500 (14)	0.0109 (13)	0.0095 (11)	0.0000 (14)
C10	0.0151 (7)	0.0161 (7)	0.0212 (7)	0.0002 (6)	0.0008 (6)	−0.0003 (6)
C11	0.0189 (7)	0.0155 (7)	0.0201 (7)	−0.0002 (6)	0.0010 (6)	0.0012 (6)
C12	0.0178 (7)	0.0216 (8)	0.0288 (8)	0.0019 (7)	0.0005 (7)	−0.0039 (7)
C13	0.0208 (8)	0.0230 (8)	0.0311 (9)	−0.0020 (7)	−0.0030 (7)	−0.0047 (7)
C14	0.0218 (8)	0.0175 (8)	0.0195 (7)	0.0009 (7)	0.0005 (6)	−0.0014 (6)
C15	0.0181 (7)	0.0151 (7)	0.0184 (7)	0.0002 (6)	0.0003 (6)	0.0009 (6)
C16	0.0190 (7)	0.0163 (7)	0.0186 (7)	−0.0009 (6)	0.0003 (6)	0.0007 (6)
C17	0.0186 (7)	0.0180 (8)	0.0207 (7)	−0.0003 (6)	0.0004 (6)	0.0033 (6)
C18	0.0210 (7)	0.0211 (8)	0.0217 (8)	0.0039 (7)	0.0046 (7)	0.0008 (7)
C19	0.0252 (8)	0.0183 (8)	0.0212 (7)	0.0025 (7)	0.0019 (7)	−0.0023 (6)
C20	0.0171 (7)	0.0270 (9)	0.0422 (10)	−0.0017 (7)	−0.0009 (8)	−0.0092 (8)
C21	0.106 (3)	0.092 (3)	0.0397 (14)	−0.069 (2)	0.0241 (16)	−0.0203 (15)

### Geometric parameters (Å, °)

**Table:**

O1—C10	1.4218 (19)	C7—C8	1.509 (3)
O1—H1O	0.8400	C7—H7	1.0000
O2—C17	1.357 (2)	C8—C9	1.309 (4)
O2—C20	1.431 (2)	C8—H8	0.9500
O3—C21	1.407 (3)	C9—H9A	0.9500
O3—H3O	0.8401	C9—H9B	0.9500
N1—C6	1.480 (3)	C10—C11	1.518 (2)
N1—C5	1.487 (2)	C10—H10	1.0000
N1—C1	1.490 (2)	C11—C12	1.372 (2)
N2—C13	1.320 (2)	C11—C15	1.424 (2)
N2—C14	1.368 (2)	C12—C13	1.412 (2)
C1—C2	1.541 (2)	C12—H12	0.9500
C1—C10	1.544 (2)	C13—H13	0.9500
C1—H1	1.0000	C14—C19	1.422 (2)
C2—C3	1.537 (3)	C14—C15	1.424 (2)
C2—H2A	0.9900	C15—C16	1.421 (2)
C2—H2B	0.9900	C16—C17	1.373 (2)
C3—C4	1.534 (3)	C16—H16	0.9500
C3—C7	1.538 (3)	C17—C18	1.426 (2)
C3—H3	1.0000	C18—C19	1.357 (3)
C4—C5	1.543 (3)	C18—H18	0.9500
C4—H4A	0.9900	C19—H19	0.9500
C4—H4B	0.9900	C20—H20A	0.9800
C5—H5A	0.9900	C20—H20B	0.9800
C5—H5B	0.9900	C20—H20C	0.9800
C6—C7	1.553 (3)	C21—H21A	0.9800
C6—H6A	0.9900	C21—H21B	0.9800
C6—H6B	0.9900	C21—H21C	0.9800
			
C10—O1—H1O	108.6	C9—C8—H8	117.5
C17—O2—C20	116.01 (13)	C7—C8—H8	117.5
C21—O3—H3O	109.2	C8—C9—H9A	120.0
C6—N1—C5	107.46 (15)	C8—C9—H9B	120.0
C6—N1—C1	111.58 (15)	H9A—C9—H9B	120.0
C5—N1—C1	107.09 (15)	O1—C10—C11	110.12 (13)
C13—N2—C14	117.82 (15)	O1—C10—C1	111.56 (13)
N1—C1—C2	111.16 (14)	C11—C10—C1	108.93 (14)
N1—C1—C10	111.80 (14)	O1—C10—H10	108.7
C2—C1—C10	113.66 (14)	C11—C10—H10	108.7
N1—C1—H1	106.6	C1—C10—H10	108.7
C2—C1—H1	106.6	C12—C11—C15	118.50 (15)
C10—C1—H1	106.6	C12—C11—C10	121.46 (15)
C3—C2—C1	107.88 (15)	C15—C11—C10	120.01 (14)
C3—C2—H2A	110.1	C11—C12—C13	119.75 (16)
C1—C2—H2A	110.1	C11—C12—H12	120.1
C3—C2—H2B	110.1	C13—C12—H12	120.1
C1—C2—H2B	110.1	N2—C13—C12	123.58 (17)
H2A—C2—H2B	108.4	N2—C13—H13	118.2
C4—C3—C2	108.53 (17)	C12—C13—H13	118.2
C4—C3—C7	108.63 (18)	N2—C14—C19	118.35 (15)
C2—C3—C7	109.48 (16)	N2—C14—C15	122.68 (15)
C4—C3—H3	110.1	C19—C14—C15	118.98 (15)
C2—C3—H3	110.1	C16—C15—C14	119.04 (15)
C7—C3—H3	110.1	C16—C15—C11	123.35 (15)
C3—C4—C5	108.25 (16)	C14—C15—C11	117.60 (15)
C3—C4—H4A	110.0	C17—C16—C15	120.28 (15)
C5—C4—H4A	110.0	C17—C16—H16	119.9
C3—C4—H4B	110.0	C15—C16—H16	119.9
C5—C4—H4B	110.0	O2—C17—C16	124.69 (16)
H4A—C4—H4B	108.4	O2—C17—C18	114.79 (15)
N1—C5—C4	111.18 (16)	C16—C17—C18	120.52 (16)
N1—C5—H5A	109.4	C19—C18—C17	120.08 (16)
C4—C5—H5A	109.4	C19—C18—H18	120.0
N1—C5—H5B	109.4	C17—C18—H18	120.0
C4—C5—H5B	109.4	C18—C19—C14	121.07 (16)
H5A—C5—H5B	108.0	C18—C19—H19	119.5
N1—C6—C7	112.16 (16)	C14—C19—H19	119.5
N1—C6—H6A	109.2	O2—C20—H20A	109.5
C7—C6—H6A	109.2	O2—C20—H20B	109.5
N1—C6—H6B	109.2	H20A—C20—H20B	109.5
C7—C6—H6B	109.2	O2—C20—H20C	109.5
H6A—C6—H6B	107.9	H20A—C20—H20C	109.5
C8—C7—C3	112.7 (2)	H20B—C20—H20C	109.5
C8—C7—C6	112.37 (19)	O3—C21—H21A	109.5
C3—C7—C6	107.13 (15)	O3—C21—H21B	109.5
C8—C7—H7	108.2	H21A—C21—H21B	109.5
C3—C7—H7	108.2	O3—C21—H21C	109.5
C6—C7—H7	108.2	H21A—C21—H21C	109.5
C9—C8—C7	124.9 (2)	H21B—C21—H21C	109.5
			
C6—N1—C1—C2	−48.65 (19)	C1—C10—C11—C12	103.71 (18)
C5—N1—C1—C2	68.68 (19)	O1—C10—C11—C15	163.18 (14)
C6—N1—C1—C10	79.54 (17)	C1—C10—C11—C15	−74.18 (19)
C5—N1—C1—C10	−163.12 (14)	C15—C11—C12—C13	2.5 (3)
N1—C1—C2—C3	−14.0 (2)	C10—C11—C12—C13	−175.43 (16)
C10—C1—C2—C3	−141.20 (16)	C14—N2—C13—C12	−2.0 (3)
C1—C2—C3—C4	−51.1 (2)	C11—C12—C13—N2	−0.1 (3)
C1—C2—C3—C7	67.3 (2)	C13—N2—C14—C19	−178.37 (17)
C2—C3—C4—C5	65.3 (2)	C13—N2—C14—C15	1.8 (3)
C7—C3—C4—C5	−53.6 (2)	N2—C14—C15—C16	−179.73 (16)
C6—N1—C5—C4	66.4 (2)	C19—C14—C15—C16	0.4 (2)
C1—N1—C5—C4	−53.6 (2)	N2—C14—C15—C11	0.6 (2)
C3—C4—C5—N1	−10.8 (2)	C19—C14—C15—C11	−179.29 (15)
C5—N1—C6—C7	−54.7 (2)	C12—C11—C15—C16	177.65 (17)
C1—N1—C6—C7	62.44 (19)	C10—C11—C15—C16	−4.4 (2)
C4—C3—C7—C8	−171.49 (17)	C12—C11—C15—C14	−2.7 (2)
C2—C3—C7—C8	70.1 (2)	C10—C11—C15—C14	175.28 (15)
C4—C3—C7—C6	64.42 (19)	C14—C15—C16—C17	−1.8 (2)
C2—C3—C7—C6	−53.9 (2)	C11—C15—C16—C17	177.88 (16)
N1—C6—C7—C8	−133.14 (19)	C20—O2—C17—C16	6.9 (2)
N1—C6—C7—C3	−8.8 (2)	C20—O2—C17—C18	−172.46 (15)
C3—C7—C8—C9	106.1 (3)	C15—C16—C17—O2	−177.66 (16)
C6—C7—C8—C9	−132.7 (3)	C15—C16—C17—C18	1.6 (2)
N1—C1—C10—O1	−76.08 (17)	O2—C17—C18—C19	179.34 (16)
C2—C1—C10—O1	50.77 (19)	C16—C17—C18—C19	0.0 (3)
N1—C1—C10—C11	162.15 (14)	C17—C18—C19—C14	−1.4 (3)
C2—C1—C10—C11	−71.00 (17)	N2—C14—C19—C18	−178.68 (17)
O1—C10—C11—C12	−18.9 (2)	C15—C14—C19—C18	1.2 (3)

### Hydrogen-bond geometry (Å, °)

**Table:**

*D*—H···*A*	*D*—H	H···*A*	*D*···*A*	*D*—H···*A*
O3—H3o···N1	0.84	1.95	2.783 (2)	171
O1—H1o···N2^i^	0.84	1.92	2.751 (2)	173
C20—H20b···O1^ii^	0.98	2.33	3.298 (2)	171
C18—H18···O3^iii^	0.95	2.58	3.471 (2)	155

Symmetry codes:  (i) −*x*, *y*+1/2, −*z*+3/2; (ii) *x*+1, *y*, *z*; (iii) −*x*+1, *y*−1/2, −*z*+3/2.
